# Rapid Remodeling of the Host Epithelial Cell Proteome by the Listeriolysin O (LLO) Pore-forming Toxin * [Fn FN2]

**DOI:** 10.1074/mcp.RA118.000767

**Published:** 2018-05-11

**Authors:** Julien Karim Malet, Francis Impens, Filipe Carvalho, Mélanie Anne Hamon, Pascale Cossart, David Ribet

**Affiliations:** From the ‡Institut Pasteur, Unité des Interactions Bactéries-Cellules, Département de Biologie Cellulaire et Infection, F-75015 Paris, France;; §INSERM, U604, F-75015 Paris, France;; ¶INRA, USC2020, F-75015 Paris, France;; ‖University Paris Diderot, Sorbonne Paris Cité, Cellule Pasteur, Paris, France;; **VIB Center for Medical Biotechnology, B-9000 Ghent, Belgium;; ‡‡Department of Biomolecular Medicine, Ghent University, B-9000 Ghent, Belgium;; §§VIB Proteomics Core, B-9000 Ghent, Belgium;; ¶¶Institut Pasteur, Unité Chromatine et infection, Paris, France

**Keywords:** Infectious disease, Host-Pathogen Interaction, Bacteria, Cell biology, Protein Degradation, Transcriptional Regulation, Post-translational modifications, Ubiquitin, SILAC, Tandem Mass Spectrometry, Listeria, Listeriolysin O, Shotgun proteomics, Toxin, Transcriptomics

## Abstract

Bacterial pathogens use various strategies to interfere with host cell functions. Among these strategies, bacteria modulate host gene transcription, thereby modifying the set of proteins synthetized by the infected cell. Bacteria can also target pre-existing host proteins and modulate their post-translational modifications or trigger their degradation. Analysis of protein levels variations in host cells during infection allows to integrate both transcriptional and post-transcriptional regulations induced by pathogens. Here, we focused on host proteome alterations induced by the toxin Listeriolysin O (LLO), secreted by the bacterial pathogen *Listeria monocytogenes.* We showed that a short-term treatment with LLO remodels the host cell proteome by specifically decreasing the abundance of 149 proteins. The same decrease in host protein levels was observed in different epithelial cell lines but not in macrophages. We show in particular that this proteome remodeling affects several ubiquitin and ubiquitin-like ligases and that LLO leads to major changes in the host ubiquitylome. Strikingly, this toxin-induced proteome remodeling involves only post-transcriptional regulations, as no modification in the transcription levels of the corresponding genes was observed. In addition, we could show that Perfringolysin O, another bacterial pore-forming toxin similar to LLO, also induces host proteome changes. Taken together, our data reveal that different bacterial pore-forming toxins induce important host proteome remodeling, that may impair epithelial cell functions.

Bacterial pathogens have developed many strategies to exploit host functions for survival, replication and escape from immune responses. A first strategy consists in interfering with host cell protein activities (1–3). Another strategy is to remodel host cell composition, for example by modifying the abundance of specific proteins. This remodeling of host cell proteome may result from deregulation of gene transcription, which involves the targeting of transcription factors or chromatin remodelers (4), or from protein degradation. Protein degradation can be achieved by targeting cellular factors to host degrading machineries such as the proteasome. Conversely, to respond to infection and to trigger anti-bacterial responses, host cells use similar processes, *i.e.* modulation of the activity of pre-existing components or remodeling of cell proteome.

Characterization of the variations in host cell protein abundance in response to infection is thus critical to understand host-pathogen interactions (5). Transcriptional profiling has been extensively used to study host cell responses to infections. mRNA concentrations are in this case used as proxies to evaluate the concentration of the corresponding proteins. In this context, it is assumed that transcript abundance correlates with protein abundance. However, it is now clear that protein abundance is strongly dependent on post-transcriptional mechanisms, which include stability of the RNA, its export rate to the cytosol, its translation efficiency by ribosomes, as well as the stability of the corresponding protein once synthetized (6). Proteomics approaches focusing on the direct quantification of proteins rather than RNA, are, in comparison, more informative as they integrate all these parameters (5).

Here, we monitored host proteome changes induced by the toxin Listeriolysin O (LLO) ^1^ secreted by the bacterial pathogen *Listeria monocytogenes. Listeria* is a Gram-positive bacterium responsible for the foodborne disease listeriosis, a leading cause of death because of food-transmitted bacterial pathogens. Although most of human infections occur by ingestion of contaminated food, some unusual cases of nosocomial infections have been reported. *Listeria* is a facultative intracellular pathogen that can infect both phagocytic and nonphagocytic cells, such as epithelial cells. In contrast to the numerous reports of global transcriptional changes induced by *Listeria* in host cells during infection (7–14), only few studies reported post-transcriptional alterations of host protein abundance (15–19). These studies focused on specific host proteins (*i.e.* UBC9, TERT, MFF, MRE11 or lysosomal proteins), and did not address whether global proteome alterations were induced by the bacterium. Interestingly, the decrease of some of these host targets, such as UBC9 or MRE11, is triggered by the pore-forming toxin LLO and was shown to be beneficial for *Listeria* infection (15, 18).

To obtain a complete picture of how the LLO toxin may impact the host cell proteome, we decided to use a combination of transcriptomic and proteomic-based approaches to monitor the expression level and the fate of host proteins in cells after exposure to the toxin. We identified a significant decrease in the levels of 149 host proteins in response to a short treatment with LLO. Strikingly, no variation in the transcription level of the corresponding genes was observed, indicating that LLO induces remodeling of the host proteome via post-transcriptional mechanisms. We identified several components of the host ubiquitin machinery as being downregulated by LLO. Consistently, we observed a massive alteration of the host ubiquitylome in response to LLO. We finally show that the alterations of protein levels detected in epithelial cells were not observed in macrophages but were similarly triggered by another cholesterol-dependent pore-forming toxin secreted by the extracellular bacterial pathogen *Clostridium perfringens*.

## EXPERIMENTAL PROCEDURES

### 

#### 

##### Cell Culture

HeLa (American Type Culture Collection (ATCC) CCL-2), Hep G2 (ATCC HB-8065) and RAW 264.7 (ATCC TIB-71) cells were cultivated at 37 °C in a 10% CO_2_ atmosphere in Minimum Essential Medium (MEM) (Invitrogen). Culture media for HeLa and Hep G2 cells were supplemented with 2 mm Glutamax (Invitrogen), 10% Fetal Bovine Serum (FBS), MEM non-essential aminoacids (Invitrogen) and 1 mm sodium pyruvate. Culture medium for RAW 264.7 was supplemented with 4 mm Glutamax, 10% FBS and 1 mm sodium pyruvate.

For stable isotope labeling by amino acids in cell culture (SILAC) (20, 21), HeLa cells were cultivated in DMEM (Dulbecco's Modified Eagle Medium) without l-lysine, l-arginine, or l-glutamine (Silantes Gmbh, Germany) and supplemented with 10% dialyzed FBS (Invitrogen), 2 mm GlutaMAX (Invitrogen), and either natural l-lysine HCl (Lys_0_) and l-arginine HCl (Arg_0_) (light labeling; Sigma-Aldrich) or D_4_ l-lysine HCl (Lys_4_), ^13^C_6_ l-lysine HCl (Lys_6_) and ^13^C_6_ l-arginine HCl (Arg_6_) (heavy labeling; Silantes Gmbh). l-Lysine HCl was added at its normal concentration in DMEM (146 mg/L), but the concentration of l-arginine HCl was reduced to 25 mg/L (30% of the normal concentration in DMEM) to prevent metabolic conversion of arginine to proline. Cells were kept for at least six population doublings to ensure complete incorporation of the labeled lysine and arginine.

##### Bacterial Strains

*Listeria* strains were grown in brain heart infusion (BHI) broth or agar plates (BD Difco) at 37 °C. Strains used in this study were *L. monocytogenes* EGD (BUG 600) and the corresponding isogenic deletion mutant EGD Δ*hly* (BUG 3650; ref. 22).

##### Bacterial Infections

For *in vitro* infections, HeLa cells were seeded at a density of 5 × 10^5^ cells per 960 mm^2^ wells the day before infection. Bacteria were cultured overnight at 37 °C, then subcultured 1:20 in BHI until exponential-phase (OD_600 nm_ of 1.0), and washed 4 times in PBS. HeLa cells were serum-starved for 2 h before infection. 4 × 10^7^ viable bacteria were added to cells (multiplicity of infection (MOI) of 50) and centrifuged on cells for 5 min at 200 × *g*. After 1 h of infection, cells were washed and harvested, or incubated for four additional hours with fresh medium supplemented with 10% FBS and 50 μg/ml gentamicin (Euromedex, France) to kill extracellular bacteria.

##### Pore-forming Toxin Treatment

Wild-type and pore-deficient LLO and PFO toxins were purified from *Escherichia coli* strains transformed with plasmids encoding hexahistidine tagged, signal peptide deficient versions of LLO and PFO, as described in (15, 22, and 23). Toxins were isolated from *E. coli* bacterial lysates using NiNTA pull-down. The degree of purification for each toxin was checked by SDS-PAGE analysis followed by Coomassie blue staining. Purified toxins were added directly in culture medium of cells serum-starved for 2 h as indicated in the text. For untreated controls, cell culture medium only was added.

##### Experimental Design and Statistical Rationale

For SILAC-based proteomic analysis, two independent biological replicates were analyzed. For label-free proteomic analysis, four independent biological replicates were analyzed. For transcriptomic analysis, RNA from three independent biological replicates were analyzed. For immunoblot analysis, cell lysates from at least two independent biological replicates were analyzed.

##### Proteomics Samples Preparation and LC-MS/MS Analysis

For the SILAC-based proteomic analyses, HeLa cells were cultivated in light or heavy SILAC media (1.2 × 10^7^ cells per SILAC condition). Cells were serum-starved for 2 h and then incubated with 3 nm of purified toxin for 20 min or left untreated. For the first SILAC experiment (experiment SILAC#1), cells grown in light medium (Lys_0_, Arg_0_) were left untreated, whereas cells grown in heavy medium (Lys_4_, Arg_6_) were incubated with LLO. Labeling was swapped for the second SILAC experiment (experiment SILAC#2): cells grown in light medium (Lys_0_, Arg_0_) were incubated with LLO, and cells grown in heavy medium (Lys_6_, Arg_6_) were left untreated. Upon treatment, cells were harvested by scraping on ice in CHAPS lysis buffer (50 mm sodium phosphate pH 7.4, 100 mm NaCl, 1% CHAPS, complete protease inhibitor tablet (Roche, Switzerland)) and lysates from differentially treated light and heavy labeled cells were mixed as described above. Mixed lysates were further subjected to 3 freeze-thaw cycles, sonicated and incubated with 2.5 U/ml of Micrococcal Nuclease I for 15 min at 37 °C. Insoluble components were removed by centrifugation for 20 min at 16,000 × *g* and guanidinium hydrochloride (Gu.HCl) was added dry to the soluble fraction to a final concentration of 2 m. Proteins were reduced and alkylated by incubation with 15 mm tris(2-carboxyethyl)phosphine (TCEP) and 30 mm iodoacetamide for 30 min at 37 °C. Proteins were desalted on disposable NAP-10 columns (GE Healthcare, UK), boiled for 5 min, put on ice for 5 min and digested overnight with sequencing-grade trypsin (Promega, enzyme/substrate of 1/100 (w/w)) at 37 °C. The resulting peptide mixture was dried completely in a vacuum concentrator, re-dissolved in 0.1% TFA, 0.5% H_2_O_2_, and incubated for 30 min at 30 °C to oxidize methionine residues. Samples were snap-frozen, dried completely in a vacuum concentrator and re-dissolved in peptide OFFGEL stock solution for fractionation into 24 fractions by isoelectric focusing using 24 cm Immobiline DryStrip pH 3–10 in an Agilent 3100 OFFGEL device, according to the instructions of the manufacturer. Fractionated peptides were purified on Omix C18 tips (Agilent), dried and re-dissolved in 20 μl loading solvent (0.1% formic acid in water/acetonitrile (98:2, v/v)) of which 10 μl was injected for LC-MS/MS analysis on an Ultimate 3000 HPLC system in-line connected to an LTQ Orbitrap Velos mass spectrometer (Thermo Scientific). Trapping was performed at 10 μl/min for 4 min in loading solvent on a PepMapTM C18 column (0.3 mm inner diameter × 5 mm (Dionex)), and following back-flushing from the trapping column, the sample was loaded on a reverse-phase column (made in-house, 75 μm I.D. × 150 mm, 3 μm beads C18 Reprosil-HD, Dr. Maisch, Germany). Peptides were eluted by a linear increase from 2 to 55% MS solvent B (0.08% formic acid in water/acetonitrile (2:8, v/v)) over 30 min at a constant flow rate of 300 nl/min. The mass spectrometer was operated in data-dependent mode, automatically switching between MS and MS/MS acquisition for the ten most abundant ion peaks per MS spectrum. Full-scan MS spectra (300–2000 m/z) were acquired at a resolution of 60,000 in the orbitrap analyzer after accumulation to a target value of 1,000,000. The ten most intense ions above a threshold value of 5000 were isolated for fragmentation by CID at a normalized collision energy of 35% in the linear ion trap (LTQ) after filling the trap at a target value of 5000 for maximum 50 ms.

For label-free proteomic analyses, 6 × 10^6^ HeLa cells were cultivated for each condition in regular culture medium. Cells were serum starved for 2 h and then incubated with 3 nm of purified LLO for 20 min or left untreated. For proteasome inhibition, cells were pre-incubated for 5h with 10 μm Lactacystin (Sigma-Aldrich) and 10 μm MG132 (Sigma-Aldrich) and then treated or not with LLO. Upon treatment, cells were harvested by scraping on ice in lysis buffer (50 mm HEPES pH 8.0, 8 m Urea, phosphatases inhibitor (PhosSTOP; Roche)). The protein concentration in the lysates was measured and 500 μg of protein from each sample material was isolated to continue the protocol. Proteins were reduced by addition of 5 mm DTT and incubation for 30 min at 55 °C and then alkylated by addition of 100 mm iodoacetamide for 15 min at room temperature in the dark. Samples were further diluted with 50 mm HEPES pH 8.0 to a final urea concentration of 4 m and proteins were digested with 5 μg LysC (FUJIFILM Wako Pure Chemical Corporation, France) (1/100, w/w) for 4 h at 37 °C. Samples were again diluted to 2 m urea and digested with 5 μg trypsin (Promega) (1/100, w/w) overnight at 37 °C. The resulting peptide mixture was acidified by addition of 1% trifluoroacetic acid (TFA) and after 15 min incubation on ice, samples were centrifuged for 15 min at 1780 × *g* at room temperature to remove insoluble components. Next, peptides were purified on SampliQ C18 columns (Agilent), dried and redissolved in loading solvent (0.1% TFA in water/acetonitrile (98:2, v/v)) and ∼2 μg of each sample was injected for LC-MS/MS analysis on an Ultimate 3000 RSLCnano system (Thermo Scientific) in-line connected to a Q Exactive HF mass spectrometer (Thermo Scientific) equipped with a Nanospray Flex Ion source (Thermo Scientific). Trapping was performed at 10 μl/min for 4 min in loading solvent on a 20 mm trapping column (made in-house, 100 μm internal diameter (I.D.), 5 μm beads, C18 Reprosil-HD, Dr. Maisch) and the sample was loaded on a 400 mm analytical column (made in-house, 75 μm I.D., 1.9 μm beads C18 Reprosil-HD, Dr. Maisch). Peptides were eluted by a nonlinear increase from 2 to 56% MS solvent B (0.1% formic acid in water/acetonitrile (2:8, v/v)) over 140 min at a constant flow rate of 250 nl/min, followed by a 15-min wash reaching 99% MS solvent B and re-equilibration with MS solvent A (0.1% formic acid in water/acetonitrile (2:8, v/v)). The column temperature was kept constant at 50 °C in a column oven (CoControl 3.3.05, Sonation, Germany). The mass spectrometer was operated in data-dependent mode, automatically switching between MS and MS/MS acquisition for the 16 most abundant ion peaks per MS spectrum. Full-scan MS spectra (375–1500 m/z) were acquired at a resolution of 60,000 in the orbitrap analyzer after accumulation to a target value of 3,000,000. The 16 most intense ions above a threshold value of 13,000 were isolated (window of 1.5 Th) for fragmentation at a normalized collision energy of 28% after filling the trap at a target value of 100,000 for maximum 80 ms. MS/MS spectra (200–2000 m/z) were acquired at a resolution of 15,000 in the orbitrap analyzer. The S-lens RF level was set at 55 and we excluded precursor ions with single and unassigned charge states from fragmentation selection.

##### Data Processing and Gene Ontology Terms Enrichment Analysis

**Fig. 1.**
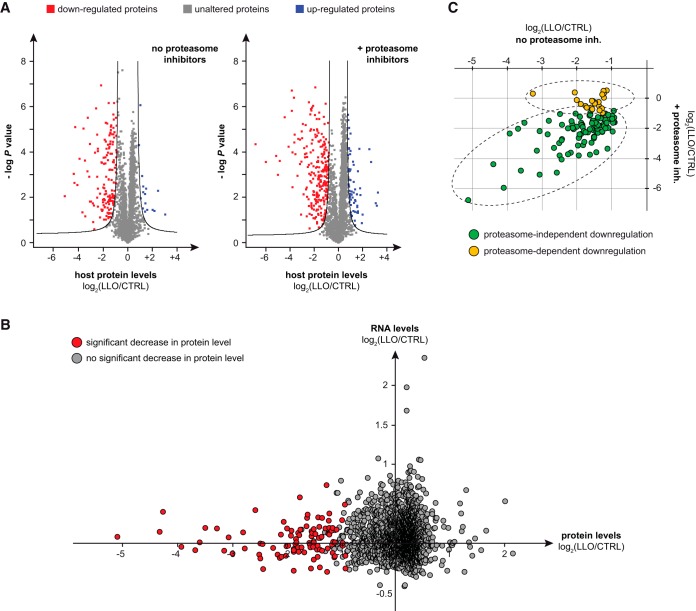
**Host proteome alterations induced by LLO.** *A*, Volcano plot showing host protein level fold changes (in log_2_) in cells treated with LLO compared with control cells (*x* axis), in the presence or absence of proteasome inhibitors. Four independent replicates were analyzed and a *t* test was performed to calculate -log *p* values for each protein (*y* axis). Black lines indicate the boundary of significance as set by the Perseus software (FDR = 0.05 and S0 = 1) (Suppl. Table S2A). *B*, Proteomic and transcriptomic analysis of HeLa cells treated with 3 nm LLO for 20 min. Each protein identified in the proteomic screen is represented by a dot, whose coordinates reflect changes in RNA and protein levels after exposure to LLO. Host factors with significant decrease in protein levels after LLO treatment are highlighted in red. *C*, Comparison of protein level changes after exposure to LLO in cells pre-treated or not with proteasome inhibitors. Each protein identified as significantly downregulated by LLO in the absence of proteasome inhibitors is represented by a dot, whose coordinates reflect protein levels changes compared with control cells (in log_2_), in the presence or absence of proteasome inhibitors (supplemental Table S2*B* and S2*C*).

Data analysis was performed with MaxQuant (version 1.5.6.5 for the SILAC analyses, version 1.6.0.16 for the label-free analyses; ref. 24) using the Andromeda search engine (25) with default search settings including a false discovery rate set at 1% on both the peptide and protein level. Spectra were searched against the human proteins in the Uniprot/Swiss-Prot database (database release version of January 2017 containing 20,172 human protein sequences for the SILAC analyses; database release version of September 2017 containing 20,237 human protein sequences for the label-free analyses, www.uniprot.org) supplemented with the sequence of listeriolysin O (RefSeq WP_003722731.1) with a mass tolerance for precursor and fragment ions of 4.5 ppm and 0.5 Da, respectively, during the main search. Enzyme specificity was set as C-terminal to arginine and lysine, also allowing cleavage at proline bonds and a maximum of two missed cleavages. Acetylation of protein N termini was set as variable modification, whereas carbamidomethyl formation of cysteine residues was set as fixed modifications. Oxidation of methionine residues was set as a variable modification in the label-free analysis and as a fixed modification in the SILAC analysis. To enable the identification of SILAC labeled peptides the multiplicity was set to two with Lys_4_/Arg_6_ or Lys_6_/Arg_6_ settings in the heavy channel, allowing for a maximum of 3 labeled amino acids per peptide. In both SILAC and label-free analyses, only proteins with at least one unique or razor peptide were retained and a minimum ratio count of two unique or razor peptides was required for quantification. From the SILAC analyses, for each protein the H/L ratio normalized by median subtraction is reported in Suppl. Table S1. For the label-free analysis, matching between runs was enabled with a matching time window of 1 min and an alignment time window of 20 min and proteins were quantified by the MaxLFQ algorithm integrated in the MaxQuant software (26). Further data analysis of the label-free samples was performed with the Perseus software (version 1.5.5.3) after loading the proteingroups file from MaxQuant. Proteins only identified by site and reverse database hits were removed and protein LFQ intensity values were log2 transformed. Replicate samples were grouped, proteins with less than three valid values in at least one group were removed, and missing values were imputed from a normal distribution around the detection limit leading to a list of 2972 quantified host proteins in the analysis with LLO treatment (supplemental Table S2*A*) and 3072 host proteins in the analysis with LLO treatment + MG132 (supplemental Table S2*B*). Next, a *t* test was performed (FDR = 0.05 and S0 = 1) to reveal proteins of which the expression level was significantly affected by LLO treatment and to generate the volcano plots depicted in Fig 1*A*. Finally, Gene Ontology terms enrichment analyses were performed using Database for Annotation, Visualization and Integrated Discovery (DAVID) bioinformatics resources (27).

##### Analysis of Host Gene Expression in Response to LLO Treatment

Total RNAs from HeLa cells treated or not with 3 nm LLO for 20 min were extracted using RNeasy kits (Qiagen, Germany). Quality of RNAs was monitored on Agilent RNA Nano LabChips (Agilent). RT on 5 μg of total RNA using oligo(dT) primers and *in vitro* transcription of the cDNA in presence of biotin were performed by using a GeneChip Amplification One-Cycle Target Labeling kit according to Affymetrix standard protocols. Fragmented, biotin-labeled cRNA samples were hybridized on Array Type GeneChip Human Genome U133 Plus 2.0. For each condition, three biological replicates were hybridized. The cell intensity files were generated with GeneChip Operating Software (GCOS). Data analysis was performed by using SPlus ArrayAnalyser software (Insightful). Statistical analysis to compare experimental condition *versus* control condition was done by using the Local Pool error test (28). The *p* values (the probability that the variability in a gene behavior observed between classes could occur by chance) were adjusted by using the Benjamini-Hochberg algorithm.

##### Antibodies

Primary antibodies used for immunoblot analysis are described in supplemental Table S3. Anti-mouse and anti-rabbit HRP-conjugated antibodies (AbCys, France) were used as secondary antibodies.

##### Immunoblot Analysis

For immunoblot analysis, cells were lysed with Laemmli buffer (125 mm Tris-HCl [pH 6.8], 4% SDS, 20% glycerol, 100 mm dithiothreitol [DTT], 0.02% bromphenol blue), boiled for 5 min, sonicated and protein content was resolved by SDS-polyacrylamide gel electrophoresis. Proteins were then transferred on PVDF membranes (GE Healthcare) and detected after incubation with specific antibodies using Pierce ECL 2 Western blotting Substrate (Fisher Scientific).

## RESULTS

### 

#### 

##### LLO Reshapes the Host Cell Proteome

To characterize host proteome alterations induced by LLO, we used shotgun proteomics on HeLa cells treated or not with the LLO toxin. We performed a first analysis using SILAC (stable isotope labeling by amino acids in cell culture) (20). The SILAC approach is based on differential isotope labeling of proteins during cell culture by metabolic incorporation of essential amino acids (predominantly lysine and arginine) that carry light or heavy isotopes. After mixing light and heavy-labeled cell lysates, proteins are subjected to trypsin digestion. The resulting peptide mixture is then separated and analyzed by tandem mass spectrometry (MS/MS). Proteins are identified by searching the recorded spectra against protein databases, and quantification is obtained by comparing light and heavy intensity for each peptide. In our experimental set up, we compared the protein content from two differentially labeled cell populations: one control population and one population incubated with a sublytic dose of LLO (3 nm) (7). Cells were exposed to LLO during only 20 min to limit protein level changes resulting from transcriptional alterations. We performed two independent experiments with swapped SILAC labeling to rule out putative labeling-dependent effects. Among the 1834 proteins that were quantified in both experiments, we identified a total of 151 proteins for which protein levels were consistently decreased in cells treated with LLO (*i.e.* with a normalized log_2_ LLO/control ratio < −0.5 in both experiments) (supplemental Fig. S1 and supplemental Table S1). These results suggest that LLO exposure triggers a remodeling of the host proteome by decreasing the protein level of many host factors.

In addition to this preliminary study, we carried out a second experiment using label-free quantitative shotgun proteomics to compare protein abundance in cells treated or not with LLO. Four independent biological replicates were included in this second screen to perform a robust statistical analysis of downregulated proteins. Degradation of UBC9, a protein known to be degraded upon LLO treatment (15), was monitored in each independent replicate using immunoblot analysis to validate LLO treatment efficiency (supplemental Fig. S2). Among the 2,972 proteins that were quantified in all independent replicates, we identified a total of 149 proteins (5.0%) for which protein levels were significantly decreased in cells treated with LLO (Fig. 1*A* and supplemental Table S2*A*). In contrast to these decreases, only 16 proteins (0.5%) showed significant increased levels in LLO-treated cells. This result confirms that LLO remodels the cell proteome mainly by decreasing the level of host targets.

We then classified proteins displaying decreased levels in response to LLO by gene ontology (GO) analysis. By looking at cellular localization, we did not observe any enrichment in proteins from specific cell compartments in the list of downregulated proteins relative to the total list of quantified proteins. This indicates that LLO-induced down-regulation affects different cellular compartments and both cytosolic and nuclear proteins. By looking at protein functions, we identified that a specific cluster of proteins, annotated “Ubl conjugation pathway” (UniProt Keywords), is significantly over-represented in the list of proteins downregulated in response to LLO (Fisher exact *p* value = 1.8 × 10^−2^). This suggests that LLO not only interferes with host SUMOylation, as previously reported (15, 29), but also targets ubiquitin and other ubiquitin-like modifications.

By focusing on proteins involved in ubiquitin conjugation, we observed that only some components were affected, suggesting a tight selection in LLO-targeted host proteins. For example, we could identify several E2 conjugases, such as UBE2B, UBE2L3, UBE2T, or UBE2V1, that are significantly downregulated in response to LLO whereas others, such as UBE2R2 or UBE2Z, remained unaffected. We furthermore observed that non-E2 components of the ubiquitin system, such as the two E1 enzymes (UBA1 and UBA6), E3 ligases (including HECTD1, HUWE1, RNF20, RNF40, RNF213, STUB1, TRIP12, UBR4, and UHRF1) and deubiquitylases (including OTULIN, OTUB1, UCHL5 and several Ubiquitin carboxy-terminal hydrolases [USP]) are unaffected by LLO (supplemental Table S2*A*). These results demonstrate that LLO-induced down-regulation affects specific components of the ubiquitylation machinery, and that LLO may thus alter only a subset of the host ubiquitylome (see below).

In addition to Ubiquitin and SUMO, we identified that the levels of two other ubiquitin-like modifiers, NEDD8 and UFM1, together with their respective E2 enzymes (UBC12/UBE2M and UFC1), decreased in response to LLO (supplemental Table S2*A*). This suggests that both neddylation and UFMylation constitute other examples of ubiquitin-like modification targeted by *Listeria* during infection.

##### The LLO-induced Host Proteome Remodeling Involves Post-transcriptional Regulations

To determine if the observed decrease in protein levels was due to transcriptional changes, we used data from a transcriptomic analysis of HeLa cells similarly treated or not with LLO for 20 min (7). By matching these transcriptomic data with our proteomics data, we could obtain information on RNA levels corresponding to 97 of the 149 proteins downregulated by LLO. Strikingly, the transcription levels of all these targets were not significantly modified in response to LLO (Fig. 1*B*). Although several genes were identified as being differentially expressed after 20 min of LLO exposure, none of them code for the proteins displaying decreased levels in LLO treated cells. This indicates that the majority of LLO-induced host protein level decreases occurs post-transcriptionally. This observation is consistent with our experimental setup, that used a short treatment with LLO (*i.e.* 20 min) and that renders unlikely variations in protein levels because of host transcription alterations.

To assess whether the host proteasome was involved in the decreased levels of these 149 proteins, we repeated our label-free quantitative proteomic analysis on HeLa cells pretreated with proteasome inhibitors before exposure to LLO. The efficiency of proteasome inhibition was validated by monitoring the increase in K48-linked polyubiquitin chains in cells treated with both MG132 and lactacystin (supplemental Fig. S2). Very interestingly, we observed that the majority (*i.e.* 83%) of host proteins identified as strongly downregulated in response to LLO also displayed significant decreased levels in the presence of proteasome inhibitors (Fig. 1*C* and supplemental Table S2*B* and S2*C*). This result demonstrates that the majority of observed decrease in host protein levels induced by LLO is not because of proteasome-mediated degradation.

**Fig. 2.**
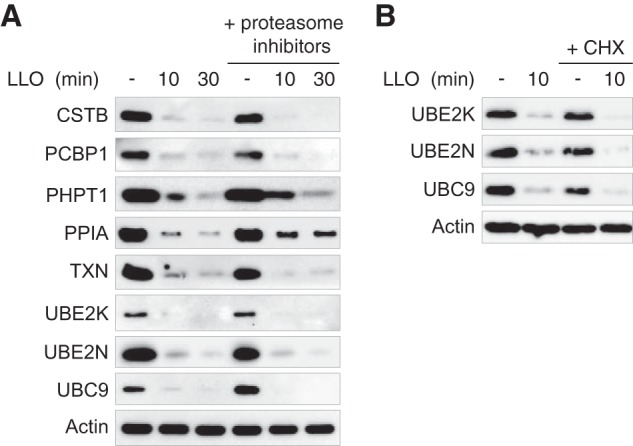
**Proteasome-independent and post-translational down-regulation of host proteins induced by LLO.** *A*, Immunoblot analysis of HeLa cells pre-treated or not with proteasome inhibitors, and then incubated with 3 nm LLO for 10 or 30 min. Antibodies against the following targets were used to monitor changes in protein levels : CSTB (Cystatin-B), PCBP1 (Poly(rC)-binding protein 1), PHPT1 (14 kDa phospho-histidine phosphatase), PPIA (Peptidyl-prolyl cis-trans isomerase A), TXN (Thioredoxin), UBE2K (Ubiquitin-conjugating enzyme E2 K), UBE2N (Ubiquitin-conjugating enzyme E2 N) and UBC9 (SUMO-conjugating enzyme UBC9). *B*, Immunoblot analysis of HeLa cells pre-treated or not with cycloheximide (CHX) for 8 h, and then incubated with 3 nm LLO for 10 min. Actin is shown as a loading control.

To further validate our proteomics data, we performed immunoblot analysis on HeLa cells treated with 3 nm LLO for 10 or 30 min and followed the protein level of several host targets identified in our different proteomic screens. We confirmed that LLO triggers a strong decrease in the level of Cystatin-B (CSTB), Poly(rC)-binding protein 1 (PCBP1), 14 kDa phosphohistidine phosphatase (PHPT1), Peptidyl-prolyl cis-trans isomerase A (Cyclophilin A; PPIA), Thioredoxin (TXN), the two E2 ubiquitin conjugases UBE2K and UBE2N and the E2 SUMO conjugase UBC9/UBE2I (Fig. 2*A*). This decrease occurs rapidly after LLO exposure (less than 10 min). In addition, we confirmed that this effect is proteasome-independent as pre-treatment with proteasome inhibitors did not block the LLO-induced decrease in the level of these host proteins (Fig. 2*A*).

Because LLO was previously reported to induce a transient inhibition of translation in host cells (30), we addressed whether the observed decreases in host protein levels were reflecting a natural decay of proteins following inhibition of translation. To this end, we blocked translation before LLO treatment by pre-incubating HeLa cells with cycloheximide (CHX) for 8 h. CHX treatment alone does not lead to a decrease in the levels of three targeted host proteins, *i.e.* UBC9, UBE2K and UBE2N, indicating that these three proteins have a half-time higher than 8 h (Fig. 2*B*). We furthermore observed a strong decrease in the levels of UBC9, UBE2K and UBE2N in cells pre-incubated with CHX and treated with LLO, definitively establishing that the decrease in the levels of these proteins occurs post-translationally (Fig. 2*B*).

**Fig. 3.**
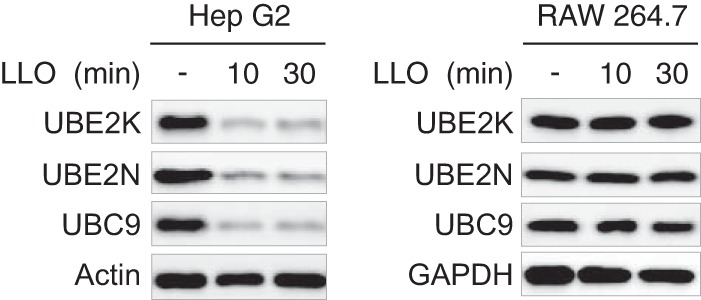
**Cell type specificity of LLO-induced down-regulation of host proteins.** Immunoblot analysis of Hep G2 and RAW 264.7 cells incubated with 3 nm LLO for 10 or 30 min. Antibodies against UBE2K (Ubiquitin-conjugating enzyme E2 K), UBE2N (Ubiquitin-conjugating enzyme E2 N) and UBC9 (SUMO-conjugating enzyme UBC9) were used to monitor changes in protein levels. Antibodies against actin and GAPDH were used as loading controls.

To extend our results to other cell lines, we treated Hep G2, a human liver epithelial cell line, and RAW 264.7, a murine macrophage-like cell line with 3 nm LLO for 10 or 30 min. Immunoblot analysis of Hep G2 cell lysates showed a strong decrease in response to LLO in the level of three targets previously identified in HeLa cells (UBC9, UBE2K, and UBE2N) (Fig. 3), indicating that LLO induces proteome alterations in different epithelial cell types. In contrast, no significant changes in UBC9, UBE2K, or UBE2N protein levels were observed in LLO-treated RAW 264.7, strongly suggesting that macrophages are resistant to, at least, some LLO-induced proteome alterations (Fig. 3).

##### Host Proteome Remodeling is Triggered During Infection by Listeria

**Fig. 4.**
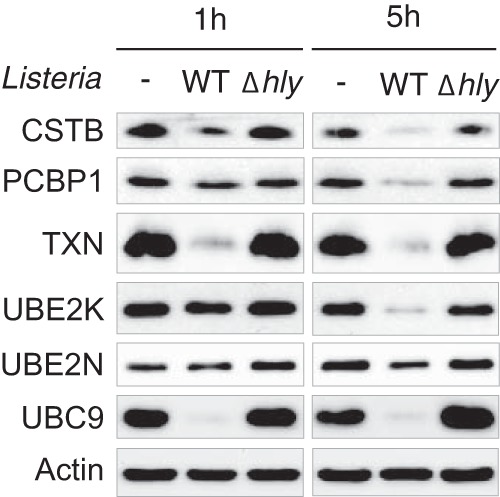
**Host proteome alterations induced by *Listeria* infection.** Immunoblot analysis of HeLa cells infected for 1 or 5h with wild-type or Δ*hly Listeria monocytogenes*. Antibodies against the following targets were used to monitor changes in protein levels : CSTB (Cystatin-B), PCBP1 (Poly(rC)-binding protein 1), TXN (Thioredoxin), UBE2K (Ubiquitin-conjugating enzyme E2 K), UBE2N (Ubiquitin-conjugating enzyme E2 N) and UBC9 (SUMO-conjugating enzyme UBC9). Actin is shown as a loading control.

To assess whether host proteome alterations observed in response to purified LLO are also induced in the context of bacterial infection, we infected HeLa cells with either wild-type *L. monocytogenes* (EGD strain), or an LLO-defective *Listeria* mutant (EGD Δ*hly*). Cells were lysed after 1h or 5h of infection and analyzed by immunoblotting experiments. We confirmed that infection with wild-type *Listeria* induces a decrease in the level of host proteins identified previously in our mass spectrometry screens (Fig. 4). This decrease was not observed during infection with a Δ*hly* strain, confirming the central role of LLO in this process (Fig. 4).

##### LLO Induces Alteration of Host Protein Ubiquitylation

**Fig. 5.**
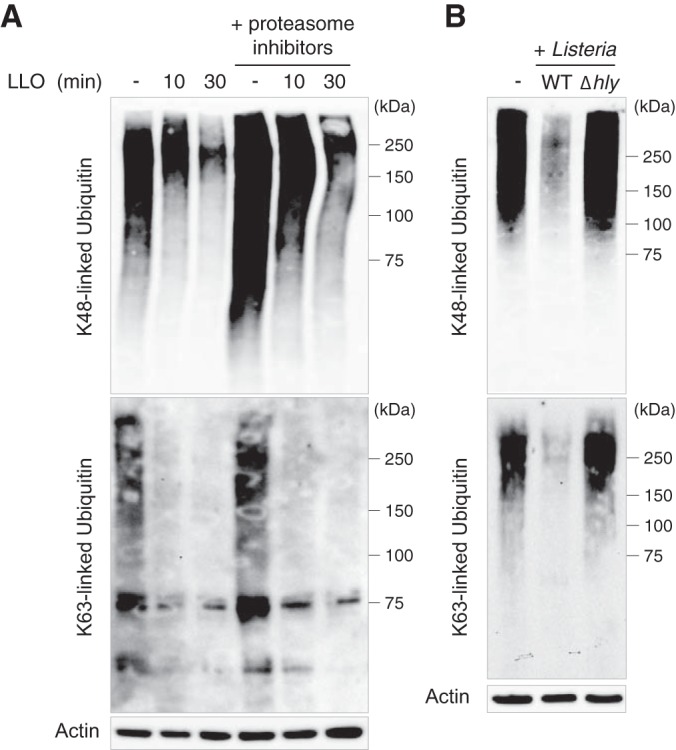
**Host ubiquitylation alterations in response to *Listeria* infection.** *A*, Immunoblot analysis of HeLa cells pre-treated or not with proteasome inhibitors, and then incubated with 3 nm LLO for 10 or 30 min. *B*, Immunoblot analysis of HeLa cells infected for 5 h with wild-type or Δ*hly Listeria monocytogenes*. Antibodies against K48- and K63-linked polyubiquitin chains were used to monitor changes in host protein ubiquitination. Actin is shown as a loading control.

As our data show that several E2 ubiquitin ligases are downregulated in response to LLO, we wondered whether this bacterial toxin alters host protein ubiquitylation. By treating HeLa cells with 3 nm LLO for 10 or 30 min, we observed, using immunoblot analysis with antibodies specific for K48- or K63-linked polyubiquitin chains, that this toxin triggers a significant decrease in the level of these polyubiquitin chains (Fig. 5*A*). Pre-treatment of HeLa cells with proteasome inhibitors does not block this effect, indicating that these decreases in K48- and K63-linked polyubiquitin chains are proteasome-independent (Fig. 5*A*). We then infected HeLa cells with wild-type *L. monocytogenes* or a Δ*hly* strain. We observed a significant decrease in K48- and K63-ubiquitylated proteins in cells infected with wild-type *Listeria* after 5h of infection, but not with the LLO-deficient mutant (Fig. 5*B*). *Listeria* infection, via LLO production, thus strongly interferes with host ubiquitylation, which corroborates the observed decrease in several E2 ligases levels.

##### Other Bacterial Pore-forming Toxins Induce Host Proteome Alteration

**Fig. 6.**
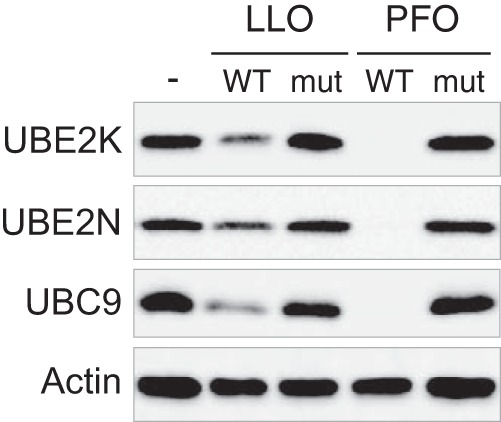
**Pore-formation dependent down-regulation of host proteins by LLO and PFO.** Immunoblot analysis of HeLa incubated for 30 min with wild-type (WT) or pore-deficient (mut) LLO and PFO toxins. Antibodies against UBE2K (Ubiquitin-conjugating enzyme E2 K), UBE2N (Ubiquitin-conjugating enzyme E2 N) and UBC9 (SUMO-conjugating enzyme UBC9) were used to monitor changes in protein levels. Actin is shown as a loading control. Wild-type toxins were used at similar hemolytic titers. Mutant toxins were used at similar protein concentration as the corresponding wild-type toxins.

We previously showed that the pore-forming toxin Perfringolysin O (PFO), secreted by the extracellular pathogen *Clostridium perfringens*, induces a decrease in the level of host UBC9, similarly to LLO (15). We here monitored whether PFO alters the level of other host proteins such as UBE2N or UBE2K. We treated HeLa cells with PFO and observed that this toxin induces a decrease in both UBE2K and UBE2N levels (Fig. 6). The decrease was not observed when cells were treated with pore formation-deficient mutants of LLO or PFO toxins (15, 22), indicating that host protein down-regulation requires plasma membrane pore formation by these toxins (Fig. 6). These results suggest that host proteome remodeling may occur in response to infection by different bacterial pathogens that secrete pore-forming toxins in the extracellular milieu.

## DISCUSSION

Within cells, the steady-state protein abundance is strongly dependent on post-transcriptional processes (6). Proteomics studies quantifying the abundance of thousands of proteins now allow researchers to integrate transcription regulation, RNA stability, translation efficiency and protein stability. By using such a proteomics approach, we quantified nearly 3000 host proteins and showed that the listerial pore-forming toxin LLO remodels the host proteome, mostly through down-regulation of protein level. Strikingly, most of protein level decreases are not because of transcriptional changes, indicating that proteome remodeling by LLO occurs at the post-transcriptional level.

The observed decrease in the abundance of 149 host proteins triggered by nanomolar concentration of LLO is a rapid process that occurs in less than 20 min. Interestingly, in this time-frame, LLO also affects the transcription of a subset of genes, but none of them code for the 149 targets identified in this study (7). Thus, exposure to LLO probably triggers several complementary waves of proteome alterations, either by directly targeting pre-existing proteins or by modulating RNA levels. In the context of *Listeria* infection, LLO-induced proteome remodeling reflects only one part of the different transcriptional and post-transcriptional changes that are triggered by this bacterium. Our results show that LLO-induced host proteome remodeling is indeed observed during infection and lasts at least for several hours. For long-term infections (*i.e.* >5 h), additional proteome alterations might be expected (combining LLO-dependent and LLO-independent remodeling), leading to a different proteomic profile of the host cells.

A previous report showed that LLO induces an arrest in protein synthesis (30). This arrest allows intoxicated cells to enter a quiescent-like state, in which minimal energy is consumed while plasma membrane damage is repaired (30). A direct consequence of this pore-forming toxin-induced arrest of protein synthesis is the disappearance of short-lived proteins (30). Here, we show that the observed decrease in the abundance of UBE2K, UBE2N and UBC9 is not because of such a translation arrest (Fig. 2*B*). This suggests that these proteins are actively degraded in response to infection, albeit in a proteasome-independent manner. Interestingly, we previously identified that LLO triggers, in epithelial cells, a permeabilization of host lysosomes and a release of lysosomal proteases such as cathepsins in the host cytosol (22). Interestingly, these lysosomal proteases are not involved in UBC9 degradation (22). Whether these lysosomal proteases are responsible for the LLO-induced degradation of some of the other cytosolic proteins identified here remains to be determined. In addition to a release of cathepsins from lysosomes, several host proteases were reported to be activated in response to LLO. In lymphocytes, LLO induces cell apoptosis, which is characterized by the activation of caspase-3 and caspase-9 (31). In macrophages, this toxin induces a calcium influx that activates a calcium-dependent calpain protease, participating to the maturation and the release of the pro-inflammatory cytokine IL-1α (32). Recombinant LLO was also shown to activate caspase-1 in macrophages and HeLa cells, via the induction of a potassium efflux and the NLRP3 inflammasome signaling, which participates in IL-1β conversion and secretion (33, 34). Finally, LLO was shown to activate caspase-7 in macrophages, which participates to the protection of the cell against the plasma membrane damages induced by the toxin (35). Whether these different host proteases, activated in response to LLO, are involved in the downregulation of the proteins identified here remains to be determined. Strikingly, we could not detect degradation products of the different LLO targets. This may indicate that protein fragments generated after the putative proteolytic cleavage of these targets are rapidly degraded by host proteases. This might also suggest that the levels of these targets decrease owing to nonproteolytic mechanisms, such as leakage outside host cells through the large pores formed by LLO (36).

Proteins identified in this study as being downregulated in response to LLO are involved in various cellular pathways. We identified several E2 ubiquitin ligases that were affected by the toxin. This result correlates with our observation of a strong alteration of the host ubiquitylome, more particularly of proteins modified by K63- and K48-linked ubiquitin chains. This result very interestingly echoes the effect of LLO on another ubiquitin-like modification, SUMOylation, where the E2 SUMO enzyme UBC9 is degraded in response to the toxin (15). As for SUMOylation, ubiquitylation is a very dynamic modification (37). The observed decrease in ubiquitylated proteins probably results from the down-regulation of E2 ubiquitin ligases, that impairs *de novo* ubiquitylation, whereas cellular deubiquitylases remain active and lead to a shift in the reaction equilibrium toward the nonubiquilyated form of the proteins. Interestingly, targeting of E2 ubiquitin ligases such as UBE2N has also been reported in the case of the intestinal pathogen *Shigella flexneri*. Indeed, this bacterium secretes an effector, OspI, that catalyzes the deamidation of UBE2N in HeLa cells. This modification blocks UBE2N activity and inhibits acute inflammatory response in the initial stage of infection (38). Thus, UBE2N might be a key host factor targeted by different pathogens during infection, albeit by different mechanisms.

In addition to E2 ubiquitin ligases, our data provide a list of other host proteins downregulated in response to LLO exposure that may play so far unknown roles during infection. Downregulation of some of these candidates may either promote infection, whereas others may dampen infection if their decrease is sensed as a danger signal by the host cell that will then trigger antibacterial responses.

Finally, we showed that PFO, another pore-forming toxin secreted by an extracellular pathogen, also triggers host proteome remodeling. In addition to LLO or PFO toxins, that both belong to the family of cholesterol-dependent cytolysins, it has been shown that the pore-forming toxin α-Hemolysin, secreted by some uropathogenic *Escherichia coli* (UPEC), also induces degradation of specific host proteins involved in cell adhesion, inflammation or cell survival (39). This indicates that host proteome remodeling triggered by pore-forming toxins is a widespread strategy used by different classes of pathogens.

Interestingly, *Chlamydia trachomatis*, an obligate intracellular bacterium, was also reported to deeply remodel the host proteome, independently from changes in transcription (40). These findings and ours highlight the importance of proteomic-based approaches that focus on protein abundance rather than mRNA levels to decipher unknown host-pathogen interactions.

## DATA AVAILABILITY

The mass spectrometry proteomics data have been deposited to the ProteomeXchange Consortium (http://proteomecentral.proteomexchange.org) via the PRIDE partner repository (41) with the dataset identifier PXD009339. Moreover, identified spectra of the label-free analysis were uploaded to MS Viewer and are accessible via following search keys: vgjqpmehba, label-free experiment LLO; owrygieix2, label-free experiment LLO + MG132.

## Supplementary Material

Supplemental Data

## Footnotes

^1^ The abbreviations used are:

LLO: listeriolysin O
PFO: perfringolysin O
SILAC: stable isotope labeling by amino acids in cell culture
CHX: cycloheximide
UBC9: SUMO-conjugating enzyme UBC9
UBE2K: Ubiquitin-conjugating enzyme E2 K
UBE2N: Ubiquitin-conjugating enzyme E2 N.
