# Supplementary material for: Rapid Remodeling of the Host Epithelial Cell Proteome by the Listeriolysin O (LLO) Pore-forming Toxin
Source: Mol Cell Proteomics. 2018 May 11;17(8):1627–36. doi: 10.1074/mcp.RA118.000767 (PMC6072537; doi:10.1074/mcp.RA118.000767)
Supplement: Supplemental Data [file supp_17_8_1627__index.html]

Rapid remodeling of the host epithelial cell proteome by the listeriolysin O pore-forming toxin — Host proteome remodeling induced by Listeriolysin O — Rapid Remodeling of the Host Epithelial Cell Proteome by the Listeriolysin O (LLO) Pore-forming Toxin — Host Proteome Remodeling Induced by Listeriolysin O — Supplemental Data 

# Rapid Remodeling of the Host Epithelial Cell Proteome by the Listeriolysin O (LLO) Pore-forming Toxin

## Supplemental Data

- Figure S1 - Supplementary Figure S1
- Figure S2 - Supplementary Figure S2
- Table S3 - Supplementary Table S3
- Supplementary Figure Legends - Supplementary Figure Legends
- Table S1-revised v2 - Supplementary Table S1
- Table S2-revised v2 - Supplementary Table S2
