# Supplementary material for: Rapid Remodeling of the Host Epithelial Cell Proteome by the Listeriolysin O (LLO) Pore-forming Toxin
Source: Mol Cell Proteomics. 2018 May 11;17(8):1627–36. doi: 10.1074/mcp.RA118.000767 (PMC6072537; doi:10.1074/mcp.RA118.000767)
Supplement: Supplemental Data [file supp_RA118.000767_137078_1_supp_132587_p87jy8.pdf]

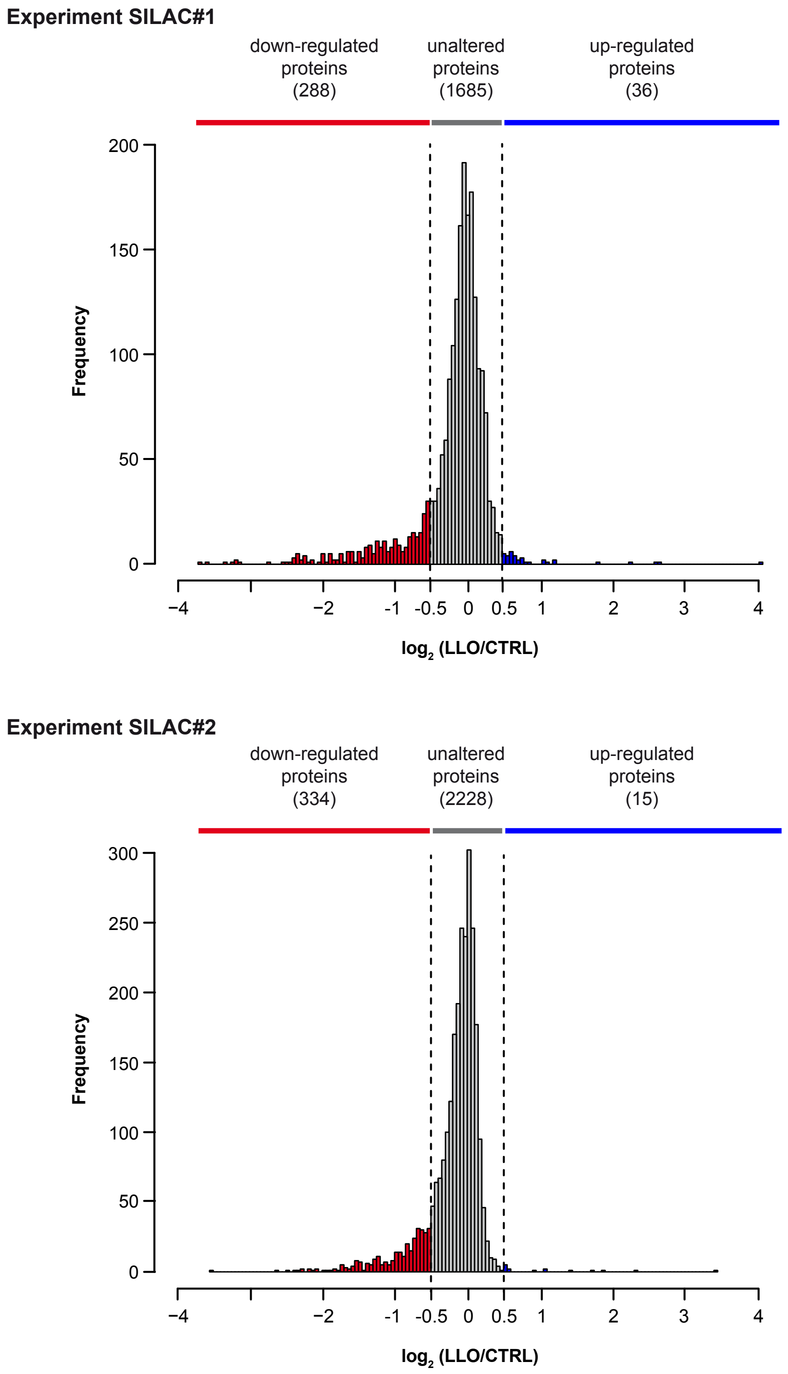

Figure S1 : SILAC-based proteomic analysis of cell exposed to LLO

SILAC-labelled HeLa cells were exposed to 3 nM LLO for 20 min (2 independent experiments). A shotgun proteomics analysis was then performed to compare proteins abundance in control (CTRL) versus LLO-treated cells. Histograms correspond to the distribution of the $\log_2$ values of the normalized LLO/CTRL ratios of all quantified proteins in each independent experiment. In both analyses, most proteins carried a $\log_2$ LLO/CTRL ratio between -0.5 and +0.5 (gray bars), indicating that their abundance is not affected by LLO. A significant fraction of proteins showed a $\log_2$ LLO/CTRL ratio < -0.5 (red bars), which corresponds to a decreased abundance in cells exposed to LLO. In contrast, only few proteins showed a $\log_2$ LLO/CTRL ratio > 0.5 (blue bars), which corresponds to an increased abundance in cells after LLO exposure.
