# Supplementary material for: Rapid Remodeling of the Host Epithelial Cell Proteome by the Listeriolysin O (LLO) Pore-forming Toxin
Source: Mol Cell Proteomics. 2018 May 11;17(8):1627–36. doi: 10.1074/mcp.RA118.000767 (PMC6072537; doi:10.1074/mcp.RA118.000767)
Supplement: Supplemental Data [file supp_RA118.000767_137078_1_supp_132583_p87jy7.pdf]

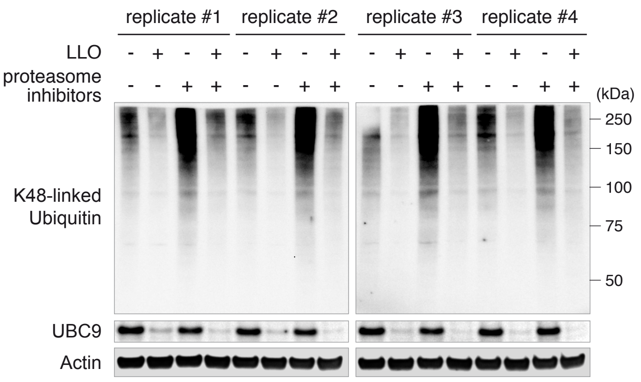

Figure S2 : Analysis of biological replicates used for label-free mass spectrometry analysis
HeLa cells, pre-treated or not with proteasome inhibitors (MG132 and lactacystin), were exposed to 3 nM LLO for 20 min (4 independent replicates), before being analyzed by quantitative label-free shotgun proteomics. Immunoblot analysis using antibodies against UBC9 and K48-linked polyubiquitin chains were performed to monitor LLO-induced downregulations and proteasome inhibition efficiencies in each independent replicate. Actin is shown as a loading control.
