# Supplementary material for: Rapid Remodeling of the Host Epithelial Cell Proteome by the Listeriolysin O (LLO) Pore-forming Toxin
Source: Mol Cell Proteomics. 2018 May 11;17(8):1627–36. doi: 10.1074/mcp.RA118.000767 (PMC6072537; doi:10.1074/mcp.RA118.000767)
Supplement: Supplemental Data [file supp_RA118.000767_137078_1_supp_132584_p87jy8.docx]

**Supplementary Table S3 : Primary antibody information.**

| **Targeted Protein** | **Dilution for Western Blot** | **Species** | **Source** | **Reference** |
| --- | --- | --- | --- | --- |
| Actin | 1:10,000 | Mouse | Sigma-Aldrich | R5441 |
| CSTB | 1:1,000 | Rabbit | Abcam | ab53725 |
| GAPDH | 1:1,000 | Mouse | Abcam | 6C5; ab8245 |
| K48-linked polyubiquitin | 1:1,000 | Rabbit | Cell Signaling Technology | D9D5; #8081 |
| K63-linked polyubiquitin | 1:1,000 | Rabbit | Cell Signaling Technology | D7A11; #5621 |
| PCBP1 | 1:1,000 | Rabbit | Abcam | ab74793 |
| PHPT1 | 1:100 | Rabbit | Abcam | ab71326 |
| PPIA | 1:1,000 | Rabbit | Abcam | ab41684 |
| TXN | 1:1,000 | Rabbit | Abcam | ab26320 |
| UBC9 | 1:1,000 | Mouse | BD Biosciences | 610478 |
| UBE2K | 1:1,000 | Rabbit | Cell Signaling Technology | #3847 |
| UBE2N | 1:1,000 | Mouse | Santa Cruz Biotechnology | F-10 ; sc-376470 |
